# Early Nutrition Programming (*in ovo* and Post-hatch Feeding) as a Strategy to Modulate Gut Health of Poultry

**DOI:** 10.3389/fvets.2019.00082

**Published:** 2019-03-21

**Authors:** Rajesh Jha, Amit Kumar Singh, Sudhir Yadav, Julio Francisco Diaz Berrocoso, Birendra Mishra

**Affiliations:** ^1^Department of Human Nutrition, Food and Animal Sciences, University of Hawaii at Manoa, Honolulu, HI, United States; ^2^Trouw Nutrition, Poultry Research Centre, Toledo, Spain

**Keywords:** broilers, gut health, histomorphology, immune system, *in ovo* feeding, post-hatch, nutritional strategy, poultry

## Abstract

Healthy gastrointestinal tract (GIT) is crucial for optimum performance, better feed efficiency, and overall health of poultry. In the past, antibiotic growth promoters (AGP) were commonly used to modulate the gut health of animals. However, considering the public health concern, the use of AGP in animal feeding is banned or regulated in several jurisdictions around the world. This necessitates the need for alternative nutritional strategies to produce healthy poultry. For that, several alternatives to AGP have been attempted with some success. However, effective modulation of the gut health parameters depends on the methods and timing of the compound being available to host animals. Routinely, the alternatives to AGP and other nutrients are provided in feed or water to poultry. However, the GIT of the newly hatched poultry is functionally immature, despite going through significant morphological, cellular, and molecular changes toward the end of incubation. Thus, early growth and development of GIT are of critical importance to enhance nutrients utilization and optimize the growth of poultry. Early nutrition programming using both *in ovo* and post-hatch feeding has been used as a means to modulate the early growth and development of GIT and found to be an effective strategy but with inconsistent results. This review summarizes the information on *in ovo* and post-hatch-feeding of different nutrients and feeds additives and their effects on gut development, histomorphology, microbiology, and immunology. Furthermore, this review will provide insight on the future of early nutrition programming as a strategy to enhance gut health, thereby improving overall health and production so that the poultry industry can benefit from this technique.

## Introduction

Poultry production has increased at a faster rate than any other livestock animal globally. Among others, the nutritionally balanced-feeding program along with antibiotic growth promoters (AGP) in poultry diets played a significant role in achieving this success. However, the poultry industry is under pressure to redefine its nutrition program to grow safe and quality meat in the light of public health concern due to the use of AGP in poultry diets. Maintenance or improvement of gut health is essential for optimum growth, better feed efficiency, and overall health of poultry (1). Also, a healthy gut is critically important for the efficient conversion of feed into absorbable form for optimal nutrient utilization, thereby better growth performance of poultry.

Gut health covers the efficient nutrient utilization, macro- and micro-structural integrity of the gut, the stability of the microbiota, and the status of the immune system (2, 3). Moreover, gut health is a complex field combining nutrition, microbiology, immunology, and physiology of animals. When gut health is compromised, digestion, and nutrient absorption are affected (3), which in turn, may have a detrimental effect on feed efficiency and greater susceptibility to diseases leading to economic loss. In addition, recent regulatory changes on the use of antibiotics, different feed requirements, and more feed-efficient animals highlight the need for a better understanding of the gut function and overall gut health. Therefore, understanding and improving gut health by different nutritional strategies are becoming a reality in the monogastric animal industries, especially when antibiotics are not allowed in food-animals feeding program (3–5).

Chicks have been shown to benefit from early access to feed and water. A healthy 1-day-old chick is a crucial link between the hatchery and the broiler farm. The delayed intake of water and nutrients to chicks could lead to a diminishing of their overall growth performance with adverse effects on breast meat. The most extreme consequence of delayed feeding is increased mortality (6). Early feeding strategies have been suggested and developed to diminish or possibly reverse the negative effects of delayed feeding. These strategies range from *in ovo* feeding to specially designed post-hatch diets (7–9). The importance of early nutrition and its effect on growth performance and different components of gut health (histomorphology, microbiota, and immune system) have already been extensively studied in the last two decades (10–15). Some studies have gone in detail about specific nutrient supplementation and its effect on the host. For example, probiotics supplementation in early life prevent pathogenic infections, amino acids (L-arginine, L-lysine, L-histidine, threonine) are beneficial in growth performance, vitamin C and E boost immunity, carbohydrates increase glycogen stores, and creatine supplement promotes muscle growth (16). Also, the *in ovo* injection of sulfur-containing amino acids (methionine plus cysteine) in the embryonated eggs exposed to heat stress have positive effects on gene expression and antioxidant indices as well as reduce the lipid profile of newly hatched broiler chicks (17). This paper has reviewed the current state of knowledge on *in ovo* and post-hatch-feeding as a strategy to enhance the gut health of poultry. It emphasizes on the effects of different nutrients on intestinal histomorphology, microbiota, and immune system using *in ovo* or post-hatch feeding system. The paper further provides the potential application of the current knowledge for the advancement of the poultry industry. Also, it has highlighted current limitations and future potential and research needs for effective use of early feeding in birds.

## Importance of Early Feeding

The perinatal period spanning from late-term embryo to few days post-hatch is an important period for the development of the gastrointestinal tract (GIT) and the immune system of poultry. Unlike mammals that can influence the development of fetus even after parturition, avian species can leverage expression only through the composition of the egg. Due to this restriction, all necessary nutrients, growth factors, and the machinery needed for the development are required to be present in the fertilized egg. Also, because of a rapidly growing embryo and the fast-metabolic turnover of modern chicks, some of the essential nutrients can become depleted or insufficient during adverse environment and disease challenges. This constraint of nutrient reserves may limit the maximal development and growth of newly hatched chicks. The availability of essential nutrients can be improved, and the existing challenges can be overcome to some extent by providing early nutrition to the embryos and chicks. Early nutrition or feeding in poultry production is a concept of providing the required nutrients to the birds either during the period when the embryo is developing or immediately after hatch until they attain a fully matured digestive system (8).

A prominent early nutrition technique that could provide further opportunity to influence the development of a chick inside the egg and overcome the constraints of nutrient limitation during late incubation phase is *in ovo* feeding (IOF). This technique provides the opportunity to supply essential nutrients, neutraceuticals, and functional foods to uplift the status of growth and development of the embryo. Several routes including amniotic (8, 18), yolk sac (19, 20), and air sac (13, 21) have been used. Amniotic fluid in the amnion surrounds the developing embryo and is believed to provide mechanical protection, prevent desiccation, and adhesion of the embryo (22). The amniotic fluid also contains protein, minerals, hormones, water, and other nutrients needed for growth and development of the developing chicks which begin to imbibe it around d 13 of incubation until internal pipping (23). This natural phenomenon of consumption of amniotic fluid by the late-term embryo toward hatch provides opportunities to add various forms of essential nutrients that would ultimately reach the GIT of chicks. Nutritional substances injected into the amniotic cavity are ingested and get deposited in the lungs and intestine due to the rhythmic respiratory movements of the late-term embryo (18, 24). *In ovo* feeding can also be used to provide adequate nutrients to the late-term embryo to protect it from the negative effect of starvation during the extended window of hatching (25). Afsarian et al. (26) found that *in ovo* injection of thyroxine along with manipulation of the eggshell temperature decreased the mortality rate occurring due to cold-induced ascites and improved chick quality and post-hatch performance. Recently, Yang et al. (27) observed that IOF of creatine pyruvate increased glucose concentration in thigh muscles of neonatal broilers, which suggests that the energy metabolism can also be altered in embryo and chicks by *in ovo* injection of different bioactive compounds. Moreover, *in ovo* application of prebiotics can be advantageous as it has been reported to increase the numbers of beneficial bacteria and promote their early colonization in the intestine of neonatal chicks (28). Likewise, investigators are interested in inoculating eggs with probiotics and synbiotics as they can be used in small amount and provide improved immunity and a better post-hatch resistance against pathogens (29, 30).

Perinatal period is also very critical as the chicks must adjust to the nature of the changing nutrition mostly from yolk-based lipid diet to carbohydrate-based solid feed. It has been observed that chicks are more efficient in utilizing lipid following hatch and gradually gain the capacity to absorb more hexoses and amino acids (31, 32). The transition of a chick from nutrient utilization from embryonic reserves to feed forces for adjustment of the newly developed digestive system. Early post-hatch feeding is essential not only for normal growth and development but also for maintaining homeostasis. Early feeding of chicks can provide readily available energy to assist in restoring hepatic glycogen stores and maintain high body temperature during initial post-embryonic days (33). In contrast, higher inclusion of anti-nutrient like non-starch polysaccharides during the early growth period can deteriorate the feed efficiency and overall productivity of growing chickens (34). Due to the practical perspective of feeding program and due to limited information on the early nutritional requirement, poultry hatchlings (e.g., chicks, ducklings, and turkey poults) are fed starter diet after hatching till 2–4weeks (34–36). Several investigators have reported that chicken weight at 6–7 weeks had the linear relationship with their weight in the first week (37) and it was not due to the breeder age and day-old chicken weight (38). The pre-starter feed could be more expensive than starter feed, but such feeding last only for a shorter period of 3–4 days and has more favorable effect on the performance of birds (39). Broiler feeding in the first few days of life is one of the priorities that could affect growth, feed efficiency, uniformity, and finally the profit of farmers. Nutrient utilization in chicks at an early stage is dependent on digestion and absorption of nutrients in the GIT (40). Improved performance has been observed in broilers by feeding pre-starter containing carbohydrate and fat during the first hours of chicken life (39, 41). Some pre-starter diets are prepared with more focus on digestible nutrients than the total requirements, and it can precondition the chick to later digest complex substrates once they acquire matured enzyme production in the GIT (7, 42). Since highly digestible alternative substrates tend to be expensive, the use of different enzymes combination or the higher activity of enzymes than those applied in later phases of diet could improve the productive performance of birds. However, limited work has been conducted to date to estimate the nutrients requirement of first week chicks that could outperform in terms of market weight and disease resistance compared with those fed starter diet (43, 44).

The GIT, especially the small intestine of poultry has the highest post**-**hatch relative growth during the first week growing period (45, 46). Therefore, an early feed deprivation can lead to a decreased intestinal enterocyte length and villus surface area which negatively affects nutrient utilization and growth (47). Early feeding is expected to influence immune development either by providing nutrients for cell proliferation and differentiation or by providing substrates for antigenic and immunomodulator activity leading to the production of several immunoglobulins (48–50). It is understood that early access to nutrients is essential for a sound immunity and improved health of chicks and poults (51). By managing a proper nutritional strategy, a specific stimulus can be generated to guide this immune system toward a more appropriate and desired direction. The requirement of enhanced immunocompetence becomes exceptionally important in the view of reducing the dependence on antibiotic growth promoters (AGPs). One of the suitable alternatives to feeding AGPs can be a supplemental chicken cytokine (52). Also, early access to feed supplemented with mannanoligosaccharides and acidifier have been reported to improve the development of intestinal morphology and immune response of chickens to *C. perfringens* challenge compared to early feed restricted chicks (10). Recently, in a study on nursery pigs, Tiwari et al. (53) reported that the use of NSP degrading enzymes modulated the production of tight junction proteins that maintain the intestinal barrier function and hence can prevent the permeability of the gut to invading pathogens. However, Mateos et al. (54) reviewed literatures on the effect of early feed access or restriction and dietary changes in the prestarter diet and concluded that the difference in the productivity tends to disappear with increasing age of birds. Still, there is a scarcity of information on early nutritional modification and most of the studies until now have focused on early access of chicks to feed. Further research is warranted to determine if early prestarter diet would be worthy of optimizing metabolic homeostasis in poultry for prospective maximal growth and feed efficiency.

## Nutrition and Gut Health in Poultry

The term “gut health” is a very comprehensive topic that requires a holistic tactic involving nutrition, gut physiology, microbiology, and immunology. Nutrition and health are interdependent, and the interface between the two occurs largely in the gut. One of the most effective ways to influence poultry gut health is through nutrition programming. In fact, the early nutrition programing by the introduction of feed and water to the chick provides a good means to feed the gut. By doing this gut, development is most favorable. This will ensure the birds are better equipped to cope with the gut challenge. There are several nutritional strategies that can be adopted to influence gut health. In this section of the review, some prominent factors affecting intestinal health are briefly presented.

### Diet Formulation

A balanced diet formulation to match nutrient content of feed with the nutrient requirements of the birds is considered as one of the most important aspects of the animal feeding program. In this respect, poultry diets should be formulated based on digestible values instead of “total” values in order to maximize protein digestibility. The ‘Balanced Protein’ (or ideal amino acid profile) concept for all the essential amino acids should be applied. The emphasis is not on minimum crude protein but rather on the proper balance or ratio of each amino acid to lysine. The use of synthetic amino acids will be beneficial and can help to prevent excess crude protein. An excess of indigestible protein in the hindgut can predispose to several intestinal challenges (e.g., wet litter).

### Starch Properties

Among the nutrients in poultry diets, starch is the most important nutrients and main source of energy in the broiler's diets (may contain up to 50% starch on a DM basis). Different cereal grains (wheat, corn, sorghum, rice) have different starch characteristics and physico-chemical properties. For example, wheat contains high levels of non-starch polysaccharides (NSP) such as β-glucans and arabinoxylans (55). The physico-chemical properties of NSP are responsible for their antinutritive activities in the broiler chicken, especially soluble viscous NSPs, which decreased the digestibility of protein, starch, and fat. In addition, high viscosity of the digesta has been shown to cause digestive and health problems, decreasing digeta passage rate, digestive enzymatic activities and nutrient digestibility, depressed feed efficiency, and growth rate of the birds (56) in diets based on barley, wheat, rye, or oats (high levels of NSP). On the other hand, insoluble and non-viscous NSPs may have a beneficial effect on gut health (57). Others NSP properties, such as resistance to the animal's digestive enzymes also promote to create a viscous environment within the intestinal lumen, resulting in excretion of sticky droppings (58). In addition, the rate of starch digestion can favor the growth of *Clostridium perfringens*. Toxins produced by *Cl. perfringens* are responsible for necrotic enteritis (10). Therefore, the application of appropriate xylanase enzymes is necessary to enhance digestion of starch and gut health because they have the ability to break down NSP and reduce digesta viscosity, increase digesta passage rate, and improve bird performance (59–61)

### Physical Texture and Form of Feed

The physical form of the raw materials used in broilers diets may affect the morphological and physiological characteristics of the entire intestinal tract (62, 63), although available published works in this area of research are inconsistent. Small grain particles give a larger surface area, and this may give rise to rapid feed passage in the gut. However, many studies (64–66) have demonstrated that larger particles promote gizzard development and activity, and, consequently, feed is retained longer in the gizzard, and its particles are more uniform when pass to the small intestine, favoring their digestion. In addition, larger particle size of feed lead to longer feed retention time in the gut; this helps to release the starch entrapped in cereal grain cells and encourage more beneficial bacterial fermentation in the caeca. Therefore, the target for particle size should be 800–1000 microns. In this respect, feeding broiler chicken with whole wheat could reduce the numbers of *Salmonella typhimurium* and *Cl. perfringens* in the GIT of the birds (63, 67). In addition, the inclusion of whole wheat into the broiler diets increased growth rate and feed efficiency of the birds (68). In contrast, Svihus et al. (69) did not find significant effects of diets containing whole wheat on body weight gain and feed efficiency. However, the authors reported that the birds fed with whole-wheat diets were more efficient and the nutrients were better digested and absorbed than the birds fed with ground wheat diets. The authors suggested that the improvements in the digestion might result from the increased pancreas and liver secretions. Svihus et al. (70) compared the inclusion of ground or whole wheat on on passage rate through the anterior GIT, and reported that although the gizzard has high capacity for processing diets with whole wheat, the average passage rate for a diet through the gizzard does not seem to be affected by the form of the wheat. Based on these results, it can be concluded that when the GIT is healthy, the inclusion of whole wheat into the diet may help to improve gut development and utilization of feed nutrients and consequently broilers performance, but when the integrity of the GIT is impaired, the inclusion of whole wheat into the diet may decrease the performance of the birds. Also, the inclusion of whole wheat in diet will reduce the energy consumption for grinding and the final feed cost of the diets.

On the other hand, use of a hammer mill to grind grains is still acceptable for the production of broiler feeds, but it may be best used with a 6-mm sieve size and a rotation speed of 750 rpm (71). A roller mill will give a more even distribution of particle size and hence better overall uniformity. In addition, a roller mill tends to produce a sharp-edged particle with less dust while a hammer mill produces a more rounded particle, which results in considerably more dust and more durable pellets. The sharper edged particles produced by roller mills may provide physical stimulation of the gut lining and therefore lead to better gut health (72).

## Effects of Early Nutrition on Histomorphology

The GIT tissue acts as a physical and immunological barrier to the harmful chemicals and infectious agents that enter the host. It also provides a path to the nutrients for proper digestion and absorption (5). Yegani and Korver (5) also found that healthy gut morphology directly affects the metabolism of nutrients, disease resistance, and immune response by the host. Diet intake exposes the GIT to the external environment whose quality, quantity and timing largely affect the delicate balance between the host, diet and gut ecology. So, feed ingredient used in the diet should be favorable to host gut structure and commensal microbiota (1). In the adverse gut environment, birds are at high risk of developing necrotic enteritis, coccidiosis, and other toxin-producing pathogens. Gut histomorphology is one of the most commonly used parameters to diagnose the status of gut health.

The effects of early nutrition on histomorphology have been studied in the last decade (13, 15, 73). It is well-documented that the first days after hatch is a critical period for the development of the mucosa because of major change occurring in the source of nutrients as the yolk is replaced by an exogenous diet. The growth of the chickens is directly linked to the digestion and absorption of nutrients, which is a result of the morphological and functional development of the small intestine. Villus height, crypt depth, and villus height/crypt depth ratio, crypt proliferation, rate of enterocyte migration, mucosal enzyme activity and goblet cell development are good indicators for the functional capacity of the intestine (74). It is well-accepted that a deeper crypt is indicative of faster tissue turnover and, perhaps, higher demand for new tissue (53, 75). Furthermore, it has been reported that a high intestinal villus is associated with a well-differentiated intestinal mucosa with high digestive and absorptive capabilities (75). A meta-analysis study done for the effect of post-hatch feed and water deprivation (PHFWD) shows significant subnormality in the small intestine segment with reduced length and relative weight of duodenum, jejunum, and ileum, and shorter villus height and crypt depth during the first week of age (74). Therefore, *in ovo* and post-hatch feeding strategies should be taken into account as a strategy to modulate the gut health of poultry.

Early nutrition is a stimulus to the early development of GIT, early absorption of the yolk sac, improved performance and the health status of the birds later in life. In the case of PHFWD, chicks have significantly lower yolk sac weight at 3 days post-hatch than on the day of hatch (74, 76). reported that *in ovo* feeding on 17–18 day of incubation is functionally equivalent to 2-day-old bird intestine with the increased size of villi and increased capacity to digest and absorb disaccharides leading to greater body weight compared to control birds fed in conventionally. However, in the commercial setup newly hatched chicks do not get access to feed and water for at least 12–36 h due to the inevitable operation of the hatchery, sex determination, vaccination, and transportation to the production farms. There are many reports on the negative effects of post-hatch fasting period on the intestine function and growth performance in the long run (77, 78). Fasting could further increase the susceptibility to infection and compromise immunity leading to an increase in the production cost of chickens (33). In the post-hatch period, there is the rapid development of intestinal length, weight, and its enzymatical activities, where delay in feeding causes a reduction in development and expression of nutrient transporters affecting absorption of nutrients (5). Apajalahti et al. (79) relate dietary factors and their interaction with a microbial profile to find out the effects on the intestinal development, mucosal architecture, and the mucus composition of the GIT. Pre- or post-hatch intestine morphological development is also very critical to digest, assimilate and absorb nutrients from the gut. Researches by Biloni et al. (80) and Adeleye et al. (77) found that fasting (feed and water) birds for 24 h negatively affects the morphology of the gut with a decrease in duodenal and jejunal villus height compared to birds fed 4-h post-hatch.

Feeding a blend of perinatal supplement and probiotic supplement shows increased villus height, villus width, villus to crypt ratio, and villus surface area along with an increase in overall body weight supported by competitive exclusion of *Salmonella* (80)*. In ovo* injection of prebiotic inulin has shown to increase the villus height on the first day and it is also known to increase mucin production to protect epithelial cells in the gut (81). Another research by Madej and Bednarczyk (82) found that *in ovo* inulin injection helps proliferate lymphoid tissue by T cells but no any effect on gut-associated lymphoid tissue. Berrocoso et al. (13) found some interesting results showing *in ovo* injection of 4.5 mg raffinose significantly affected the CD3 and ChB6 genes which are associated with the activity of T cell and B cell. The authors also found a linear increase in villus height and villus to crypt ratio of post-hatch birds with the increase in dose of raffinose. *In ovo* injection prebiotic galacto-oligosaccharides increased the overall body weight of broilers on 5th week due to its activity on sodium-dependent glucose co-transporters in the gut helps in monosaccharides absorption (83). The authors also found an increase in pancreatic secretion of trypsin and amylase on embryonic day 21 and day 7 post-hatch. *In ovo* injection of synbiotic preparation increases the number of goblet cells in the jejunum and ileum (84). These goblet cells produce mucus and protect the gut epithelium. The author also found an increase in width of the duodenal villi and crypt depth of 21-day old chickens fed *Laminaria spp*. as *in ovo* preparation. In conclusion, we can claim that early dietary intervention impact on all aspect of gut health such as gut microbial ecology, gut epithelium and immune system leading to benefit the overall growth performance of the chickens.

## Effects of Early Nutrition on Gut Microbial Ecology

The confounding performance of modern chicken to gain body weight by 25% in a day by newly hatched chick and 5,000% by 5 weeks to 2 kg makes it more demanding to meet the nutrient requirement along with the enhancement of the microbial function to improve immunity, digestive and absorptive function to overall improvement of the bird growth (2). The established protective intestinal microbiota is very stable, but it can be influenced by different dietary, disease, and environmental factors. For example, feed additives (antibiotics, coccidiostats, buffers, or acidifiers that influence gut pH), disease and hygiene conditions (clean vs. dirty environment, pathogen load in the feed ingredients, humidity of the shed, litter type, and usage), and stress (change of feed, sudden disturbances, heat, or water stress) could also affect gut microflora. However, diet is perhaps the most critical factor influencing the gut microflora (1, 85). Young animals are more affected by dietary manipulations such as its composition, processing, digestibility, and feeding method may disturb the balance in the gut ecosystem, especially in young animals (79, 86–89). For example, corn and sorghum-based diet increased *Enterococcus*, barley-based diet increased *Lactobacillus*, oat increased *Escherichia* and *Lactococcus*, and rye-based diet increased the *Streptococcus* (90). Researches have shown that the gut immunity changes with the changes in the gut microbial activity. Newly hatched chickens are very prone to colonize their gut by pathogenic bacteria as they provide near-sterile and suitable environment and continues to establish a relatively stable ecology with the animal age. Here interaction between microbiota and the host plays a deciding role to determine the future ecology and stability of such microbiota in the gut. Pourabedin and Zhao (91) explained this shift of Clostridiaeae and Enterobacteriaceae as dominant ileum microbiota families on day 7 to Lactobacillaceae and Clostridiacea on day 35. Apajalahti et al. (92) showed that bacterial densities one-day post-hatch in the ileum and cecum of the broiler reaches 10^8^ and 10^10^ cells per gram of digesta, respectively, and attends optimum levels of 10^9^ and 10^11^ per gram of ileal and cecal digesta, respectively, by 3 day post-hatch. This bacterial density remains stable for further 30 days or so in broiler chicken. Supplementing prebiotics in the diet at an early stage of life increases the abundance of *Lactobacilli* and *Bifidobacterial* and suppress *coliform* (93). Whereas, a study by Villaluenga et al. (94) found that *in ovo* administration of symbiotic on day 12 or 17 of incubation, increased the number of bifidobacteria in the post-hatch period. Another study by De Oliveira et al. (29) injecting *in ovo* probiotic preparation of *Enterococcus faecium* bacteria reduced the number of *Salmonella enteritidis* positive chicks post-hatch. This indicates that *in ovo* colonization of probiotics is potential enough to post hatch bacterial infection. In general, prebiotics stimulates the microbiome when administered *in ovo* at the early age of around 12 days and later probiotic at 17/18 days of incubation causes competitive exclusion. Thus, early beneficial microbiota colonization is very critical for the proper growth of chickens (80). Many hi-tech molecular techniques are used to study the gut microbiota; high-throughput sequencing is the most common one, along with targeted amplicon sequencing followed by shotgun sequencing, metaproteomics. These techniques have some limitations regarding characterizing the functional activity of gut microbiota, thus the holistic approach using multiomics might help to better understand these gut microbiota (85).

## Effects of Early Nutrition on the Immune System

Since there is a very short time for the chicks to grow to a marketable age, it becomes essential to adopt a sound management practice that would not only ensure a general well-being of birds but would also assist them to maintain a healthy gut microbiome, strong immunity and improved gut health. Several nutrients are important in the early development of the immune system. Vitamin A is necessary to maximize immuno-competence and for the optimum growth and feed efficiency of poultry (95, 96). Other nutrients which can affect early immune development are linoleic acid, iron, selenium, and some of the B vitamins (97). Much development of immune tissues in poultry occurs at late incubation and early post-hatch period. Thus, maternal nutritional status and deposition of nutrients as well as early nutrition play an important role in the modulation of the nutritional immune system. It has been known that vitamins A, D, and E have regulatory roles in the immune system (98). The complexity of the immune response requires various modes of communication of immune cells and immune-molecules. It has been found that the poultry is most susceptible to the invading pathogens during early hatch period as their immune system does not attain the full functionality by this age.

Nutritional modulation by providing micronutrients and required substrates through both *in ovo* and in feed during first-week post-hatch can support the proliferation of lymphoid organs and modify the population of the microbiome in GIT (97). This can ultimately result in improved immunity and enhanced integrity of the intestinal epithelium of growing birds.

The intestinal mucosa is exposed to a variety of non-self external materials including pathogenic microbes and its gut-associated lymphoid tissue (GALT) plays a significant role in the avian immune system (99). Hence, it is critical that delay in feeding is avoided as it can delay the onset of GALT function (11). *In ovo* injection of nutrients can play a vital role where delay in feeding is expected due to various limitation arising due to shipping and distribution of the day-old chicks.

*In ovo* manipulation of the embryo is not only a method of filling up the reserves with nutrients but it is also a tool that allows injecting other substances that can stimulate the immune system, modify the gut microbiome, and shift the level of production of metabolites. Dibner et al. (51) proposed the effect of early oral nutrition on the development of the immune system in hatchlings broiler chicks. According to the authors, early nutrition can provide the limiting substrates, affect endogenous levels of hormones or immunomodulators, and the presence of antigen in GIT can trigger the complete differentiation of immune cells like B-lymphocytes. Recently, the focus has been intensified on nutritional manipulation of growing embryo for improvement in immunity and health of birds during later growth periods. The improvement in the immune status of poultry by use of vitamins, amino acids, and prebiotics through *in ovo* feeding has been encouraging (13, 100, 101). Similar to the late *in ovo* feeding, Kadam et al. (102) injected amino acid threonine into the yolk sac of 14 d old embryo and found that it improved the humoral response of the broiler chicks. Bakyaraj et al. (103) reported that *in ovo* injection of amino acids, trace elements, and fatty acids and vitamins into the amniotic cavity of late-term embryo improved the cell-mediated immunity in chicks. A research conducted by Selvaraj and Cherian (104) using *in ovo* injection of fatty acids into amniotic cavity revealed that injection of linoleic acid (ω-6 fatty acid) increased cell-mediated immunity while the injection of ω-3 fatty acids of marine origin induced a humoral response. Likewise, glucose triggered humoral immunity while fructose and ribose modulated cellular immunity in broilers receiving *in ovo* injection in yolk sac/amnion on d 14 of incubation (105). It has also been found that *in ovo* feeding of amino acids can enhance the growth-related genes and modulate the expression of immune genes in broilers (106). Early improvement in the status of immunity in broilers is also dependent on the general health status and nutritional state of the bird. Some substrates like mannanoligosaccharides have the potential to exert a direct effect on the maturation of enterocytes, enhancement of digestive capacity and improvement of epithelial barrier function when fed *in ovo* (107). Also, early feeding of lectin extract into the amniotic cavity of chicken in a study by Dalloul et al. (108), produced resistance against orally challenged coccidiosis. This protection of chicken against coccidiosis infection is a clear indication of immunopotentiating effect of nutritional programming. The nitric oxide (NO) is produced by cells involved directly or indirectly in immune response and has a key role in immune regulation and neurotransmission. Besides its role as a signal molecule, NO can also act as a non-specific component of the immune system by destroying the invading pathogens, tumor cells, and parasites. *In ovo* feeding of arginine to late-term embryo has been documented to increase secretory immunoglobulin A (sIgA) in the intestinal mucosa and activate Arg-NO signaling pathway in broilers (109). The increase in the level of NO and sIgA indicates that *in ovo* feeding of arginine can enhance the immunological barrier function of intestinal mucosa and improve the overall immunity and intestinal health of birds.

The application of some additives as a nutritional approach to tackle deficiencies during the early feeding period also have some prospect in reinforcing the immunity of growing poults. Feeding of β-glucan can strengthen the innate immunity by up-regulating the oxidative burst, phagocytosis and bactericidal killing capacity of heterophils and decrease the incidence of organ invasion by *Salmonella enteritica* in neonatal chickens (110). Early feeding of the amino acid is essential as their oxidation rate increases during inflammation, and a regular diet may not accommodate the requirement of growing and challenged birds. It is known that glutamine provides energy and nitrogen source for the proliferation of immune and intestinal mucosal cells and is required along with cysteine for the synthesis of antioxidants like glutathione (111, 112). However, it is suggested that breeding poultry for higher immunocompetence would have some negative impact on some production performances that is not desirable in modern poultry production due to the short time of feed to meat or egg turnover (113). Hence, further works are required to be conducted to ascertain the effect of early nutritional programming vs. delayed intervention for tackling challenges originating due to metabolic and infectious diseases in poultry production.

## Future Potential and Limitations of Early Nutrition Programing

The continuous development and improvement of *in ovo* technology have established a new scope for perinatal nutrition, allowing and creating new challenges and opportunities for nutritionists to optimize poultry production. The *in ovo* injection of important nutrients or substances into the amnion is a novel way to feed critical dietary components to embryos. Indeed, *in ovo* feeding may “jump-start” development, improving the nutritional status of the perinatal chick. The *in ovo* feeding technique has several advantages, including improvements of total digestive tract capacity; increased body weight, growth rate, and feed efficiency; reduction of post-hatch mortality and morbidity; improvements in the immune system and the response to enteric antigens; reduction in incidence of developmental skeletal disorders; and increase in muscle development and breast meat yield. The next step in the early nutrition could be to imprint genes of a bird at a very early age and turn it into a more efficient animal later. In addition, the administration of digestible nutrients into the amnion of embryos can bring an improvement in bird quality, increased glycogen reserves, fast development of the total digestive tract superior skeletal health, better muscle growth rate, higher body weight gain, improved feed conversion, and enhanced immune function (16). Using nutrigenomic data, almost 30 percent of genes expressed different activity over time by *in ovo* feeding.

The main limitations still are associated with embryo development and nutrient metabolism. Another question is a limitation in the probiotic preparation that fits the specific needs of the individual bird. Future early nutrition would be feeding complex symbiotic that would replace feed additives and supplements in the post-hatch feed and is more beneficial to the overall poultry industry.

## Conclusion

With the increase in productivity and highly feed efficient birds, the nutritional demand of embryos and early aged chicks has changed over decades. Early nutrition programing is one of the latest and successful methods to feed embryos and recently hatched chicks to prepare chickens with the healthy gut, favorable microbiota, improved immunity, and overall improved growth performance. Currently used materials to feed as early nutrition includes probiotics, prebiotics, exogenous enzymes, amino acids, hormones, vaccines, and drugs. Early feeding to chicks with these nutrients and supplements has been found to improve total digestive tract development, increase growth rate and feed efficiency, reduce post-hatch mortality and morbidity, promote growth of beneficial gut microbiota, improve the immune system and the response to enteric antigens, reduce incidence of developmental skeletal disorders, and increase in muscle development and breast meat yield. Further works are required to fine-tune the *in ovo* feeding technique for application at commercial scale in farm condition, understand the embryonic development and nutrient metabolism process more precisely, and understand how early nutrition affects specific genes responsible for performance, intestinal health, and overall health-related traits in poultry.

## Author Contributions

AS, SY, and JB wrote this review manuscript. BM reviewed the manuscript and provided critical suggestions and comments. RJ decided a review topic, reviewed the literature and this review manuscript and provided critical review, suggestions, and comments.

### Conflict of Interest Statement

The authors declare that the research was conducted in the absence of any commercial or financial relationships that could be construed as a potential conflict of interest.
